# Supplementary material for: Cloning, Expression and Evaluation of Thioredoxin Peroxidase-1 Antigen for the Serological Diagnosis of Schistosoma mekongi Human Infection
Source: Diagnostics (Basel). 2022 Dec 7;12(12):3077. doi: 10.3390/diagnostics12123077 (PMC9777376; doi:10.3390/diagnostics12123077)
Supplement: Supplementary file 1 [file diagnostics-12-03077-s001.zip › diagnostics-2072227-supplementary.pdf]

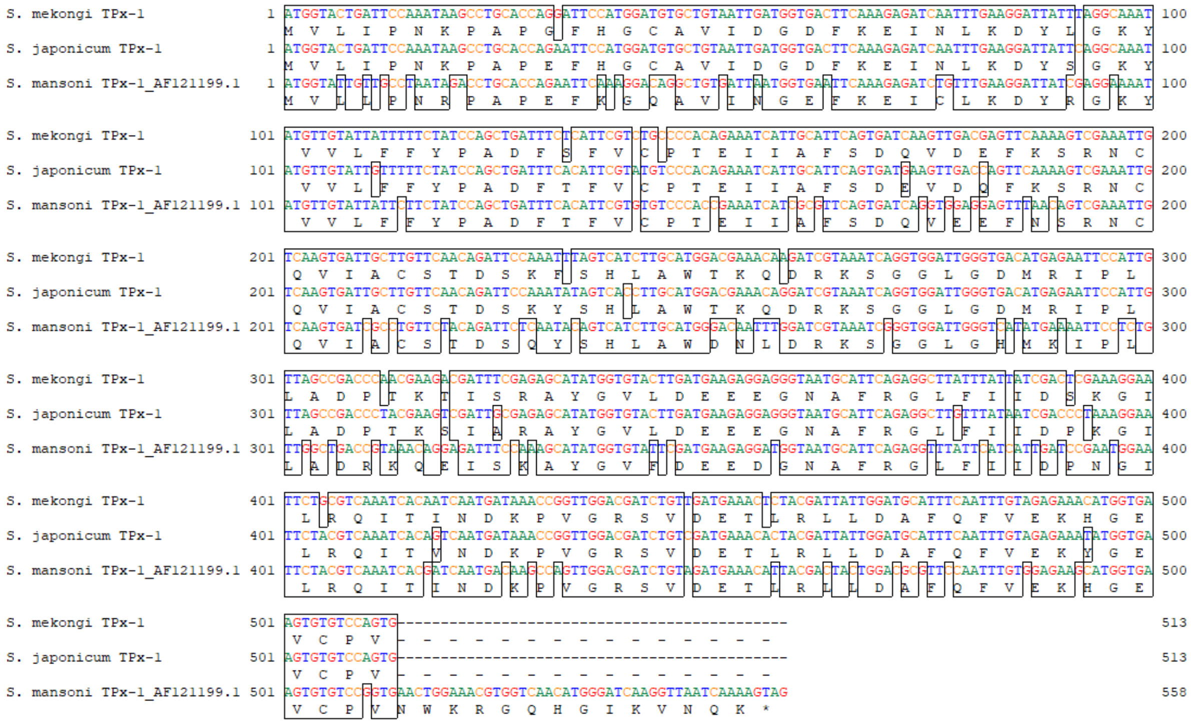

Figure S1. Alignment of nucleotide and amino acid sequences of *Schistosoma mekongi* TPx-1with *S. japaonicum* TPx-1 (AB126036.2) and *S. mansoni* TPx-1 (AF121199.1). The alignment was obtained by ClustalW. Identical nucleotides and residues between the sequences are boxed.

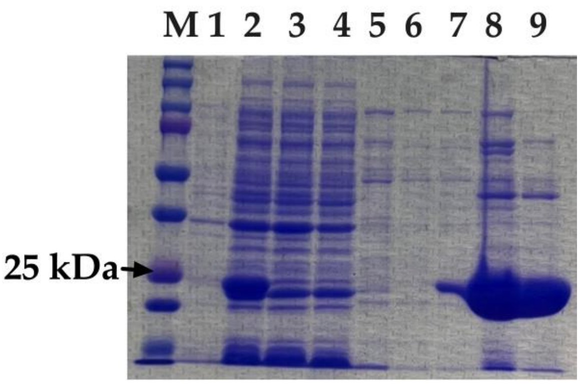

Figure S2. The expression and purification of SmekTPx-1 confirmed through SDS-PAGE. Lane M: marker, lane 1: *E. coli* culture before adding IPTG, lane 2: *E. coli* culture after adding IPTG, lane 3 *E. coli* culture after sonicating, lane 4: Flow-through the column, lane 5: Wash buffer from the first washing, 6: Wash buffer from the second washing, 7-9 Protein fraction obtained in the first, second and third elution, respectively (see Materials and Methods). The arrow indicates rSmekTPx-1 with the expected molecular weight of 22kDa.

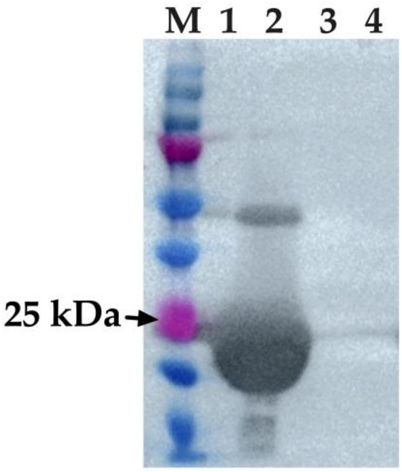

Figure S3. The expression and purification of SmekTPx-1 confirmed through Western blotting. The recombinant protein was evaluated by 12% of SDS-PAGE and was transferred onto a polyvinylidene difluoride (PVDF) membranes. Membranes were blocked at 4°C for overnight with 3% skimmed milk in blocking buffer (Tris-buffered saline with 0.05% of Tween 20 (TBS-T)). Subsequently, membranes were probed with anti-6XHis Tag mouse monoclonal antibody (Invitrogen) at 1:1000 dilutions in blocking buffer at 37°C for 1 hour. The membranes were washed three times with TBS-T and was incubated with an anti-mouse IgG conjugated with horseradish peroxidase (HRP) (GE Healthcare, IL, U. S. A.) at 1:10000 dilutions and incubated at 37°C for 1 hour. Lastly, the specific antigen-antibody reaction was detected using the ECL Western Blotting Detection Reagents (GE Healthcare) and chemiluminescence was observed using ImageQuant LAS 500 machine (GE Electric, Tokyo, Japan). Lane M: marker, lane 1-4: Protein fraction obtained in the first, second, third and fourth elution with the elution buffer, respectively (see Material and Methods). The arrow indicates the rSmekTPx-1 with the expected molecular weight of 22kDa. Overloading of the sample causes its downward spread in Lane 2.
